# A Network Structure Entropy Considering Series-Parallel Structures

**DOI:** 10.3390/e24070852

**Published:** 2022-06-21

**Authors:** Meng Cai, Jiaqi Liu, Ying Cui

**Affiliations:** 1 School of Humanities and Social Sciences, Xi’an Jiaotong University, Xi’an 710049, China; liujiaqi@stu.xjtu.edu.cn; 2 School of Mechano-Electronic Engineering, Xidian University, Xi’an 710071, China; ycui@xidian.edu.cn

**Keywords:** complex network, entropy, series-parallel structure, heterogeneity, consulting network

## Abstract

Entropy is an important indicator to measure network heterogeneity. We propose a new network structure entropy, SP (series-parallel) structure entropy, based on the global network topology while adding a medial measure that considers the series-parallel structure. First, the results of special networks show that SP structure entropy can overcome other structure’s entropy deficiencies to some extent. Then, through simulation analysis of typical networks, the validity and applicability of SP structure entropy in describing general networks are verified. Finally, we analyze an enterprise consulting network to demonstrate the superiority of the SP structure entropy for real network analysis.

## 1. Introduction

A complex network is an abstraction of a complex system, and this is one of the essential means to quantitatively describe a complex natural system and social system and to then conduct research in fields, including virus transmission control [1,2,3], disaster spread control [4,5], financial market analysis [6], structural efficiency evaluation [7] and evolutionary game simulation [8]. Although the various parts of a complex network are interrelated, they show a high degree of heterogeneity in connection mode and that the structural characteristics of heterogeneity can greatly impact the network function [9]. Therefore, network heterogeneity has received significant attention from scholars.

Among these, entropy, as a measurement of the degree of system disorder, is widely used due to its unique connotation and penetration and has become an important indicator to measure network heterogeneity [10]: The macroscopic meaning of entropy is a measure of the uniformity of system energy distribution [11]. The stronger the heterogeneity, the smaller the entropy value; conversely, the greater the entropy value. At present, many studies have defined network structure entropy from different angles [12,13,14,15,16]. However, the form of network entropy used has always been the focus of complex network research.

In existing studies, network entropy is mainly defined from two aspects: information theory and network structure characteristics. On the one hand, in the network entropy based on information theory, Costa et al. proposed search information entropy, target entropy and information path entropy, which are defined by the information search capability of the network [17]; Boccaletti et al. defined the Shannon entropy of the network through the information theory of network topology [18].

On the other hand, in the network entropy, which focuses on reflecting network structure characteristics, Wang et al. and Wu et al., respectively, defined network entropy in terms of the difference of network nodes and degree distribution entropy [19] and Wu structure entropy [12]. Cai et al. argued that the structure entropies based on degree distribution and degree relative value are not fully considered when describing network characteristics [14] and thus proposed a network structure entropy based on point and edge difference (SD structure entropy) and a network structure entropy based on maximum flow (FB structure entropy).

Degree distribution entropy defines network structure entropy according to the importance of network nodes [19], which depends on the relative number of nodes with the same degree value in the network, $H=-\sum_{k=1}^{N}p\left(k\right)\log p\left(k\right)$, where p(k) is the distribution function for a network node degree of *k* and *N* is the number of nodes in the network. Wu structure entropy measures the network heterogeneity using the relative degree value of a node, and this is defined as $H=-\sum_{i=1}^{N}\frac{k_{i}}{\sum_{i=1}^{N}k_{i}}\log\left(\frac{k_{i}}{\sum_{i=1}^{N}k_{i}}\right)$, where $k_{i}$ is the degree of node *i*.

The above two network structure entropies only take neighbor nodes as consideration objects. Still, they do not consider the influence of non-neighbor nodes, which cannot reflect the global description of the network. In addition, because the degree distribution entropy and Wu structure entropy consider network structural characteristics in terms of “nodes” or “edges”, they have limitations in describing the heterogeneity of the special network, such as a star network [14]. For example, a star network shows weak heterogeneity under the description of degree distribution entropy, while Wu structure entropy has difficulty in reflecting the scale effect of a star network.

Cai et al. proposed a network structure entropy based on the difference between nodes and edges–SD structure entropy [14]. The node difference of node *i* is defined by the number of nodes in the network that are different from the degree value of node *i*, $S_{i}=\left[1-p\left(k_{i}\right)\right]N$, and the edge difference is defined as $D_{i}=k_{i}\left[1-p\left(k_{i}\right)\right]N$, where $k_{i}$ is the degree of node *i* in the network and $p(k_{i})$ is the probability of nodes with degree $k_{i}$ in the network. Considering the node difference and the edge difference, the structural importance of node *i* is $I_{i}^{\prime }=\alpha S_{i}+\beta D_{i}$, and the network structure entropy is defined as $H=-\sum_{i=1}^{N}\frac{I_{i}^{\prime }}{\sum_{j=1}^{N}I_{j}^{\prime }}\log\left(\frac{I_{i}^{\prime }}{\sum_{j=1}^{N}I_{j}^{\prime }}\right)$.

Compared with the degree distribution entropy and Wu structure entropy, SD structure entropy unifies the node and edge as a measurement standard. However, the essence is still starting from the degree value of nodes, emphasizing too much the network local characteristics and ignoring the global topological structure of the network. To comprehensively evaluate the local and global characteristics of the network, Cai et al. introduced the concept of network flow into the definition of structure entropy, combined radial and medial measures, and proposed a maximum flow-based network structure entropy–FB structure entropy [14].

The medial measure is the absolute flow betweenness of node *k*, $b_{k}^{\prime }=\sum_{i,j\in S(k)}{(}^{k}W_{i,j}{-}^{k}W_{i,j}^{*})$, S(k)={(i,j):1≤i≤N;1≤j≤N;i≠j≠k}. This measures the change of network flow after a node in the network removes or stops transmission, where *W* denotes the maximum flow matrix between all node pairs in the network; ${}^{k}W$ denotes the *k*th row and *k*th column removed from the matrix *W*; ${}^{k}W^{*}$ denotes the maximum flow matrix recalculated after the removal of node *k* from the original network; and the radial measure $d_{k}^{\prime }$ is the node *k* degree value, which reflects the connection between a given node and other nodes in the network.

Combining medial and radial measures, the structural importance of node *k* is defined as $I_{k}^{\prime }=\alpha b_{k}^{\prime }+\beta d_{k}^{\prime }$, and the FB structure entropy is $H=-\sum_{k=1}^{N}\frac{I_{k}^{\prime }}{\sum_{n=1}^{N}I_{n}^{\prime }}\log\left(\frac{I_{k}^{\prime }}{\sum_{n=1}^{N}I_{n}^{\prime }}\right)$, where α and β are the weights of the two measures, respectively. Compared with the first three types of network structure entropy, FB structure entropy can measure the network heterogeneity from a global perspective and reflect the differences in topology of network connections more carefully.

From degree distribution entropy and Wu structure entropy to SD structure entropy and then to FB structure entropy, the definition of network structure entropy changes from considering a “node” or “edge” unilaterally to considering both “node” and “edge” and then introducing the maximum flow to describe the network structure globally. The ability to characterize the network heterogeneity has been enhanced; however, ultimately, they have limitations.

Degree distribution entropy and Wu structure entropy only consider network structure characteristics from the “node” or “edge”, resulting in certain problems in describing the heterogeneity of special networks, such as star networks [14]. SD structure entropy fully considers the node difference and edge difference and has a good explanation for the heterogeneity of sparse network and star network; however, it is still based on the node degree value, with too much emphasis on the local characteristics of the network and then ignores the global characteristics of the network [14].

FB structure entropy introduces the concept of network flow, describes network heterogeneity from global structure, which—to an extent—overcomes the shortcomings of the other three structure entropies in reflecting the network heterogeneity; however, the network maximum flow may be limited by the maximum flow of a certain path.

**Figure 1:**
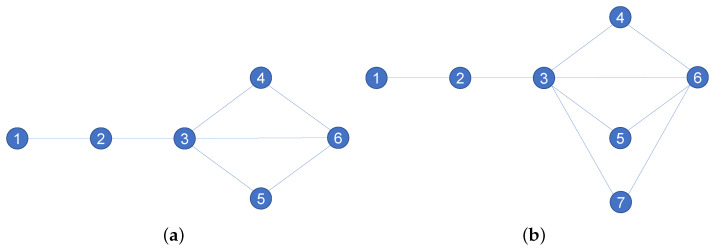
A set of 0-1 networks with a series-parallel structure given source node 1 and sink node 6, where the set of nodes in (**a**) is 1,2,3,4,5,6 and (**b**) adds node 7 to (**a**).

Under this restriction, even if the network topology is changed, the FB structure entropy cannot reflect the change in network heterogeneity. For example, Figure 1 shows a set of 0-1 networks with a series-parallel structure given source node 1 and sink node 6. It can be seen that Figure 1a has three parallel paths between node pairs (3, 6), and Figure 1b adds node 7 to Figure 1a. In Figure 1a, node 2 and node 4 are in completely different positions in the network structure, with the former not in any parallel structure and the latter in the parallel structure of node pair (3,6), and thus their importance in the network is different.

However, degree distribution entropy, Wu structure entropy and SD structure entropy are unable to distinguish the difference because their essence is to calculate the importance of a single node based on the local structure of the network. Furthermore, compared to Figure 1a, Figure 1b adds a parallel path (3, 7, 6) between the node pair (3, 6), and the importance of nodes 4 and 5 in the parallel structure is shared and thus changes. However, path (1, 2, 3) in the 0-1 network becomes the maximum flow bottleneck from node 1 to node 6, and the maximum flow completely depends on the path set {(3, 4, 6), (3, 6), (3, 5, 6), (3, 7, 6)}; yet, since each path in the set is a possible maximum flow path, the maximum flow does not depend on any one of them, and the FB structure entropy cannot reflect the change of network heterogeneity.

Aiming at the limitations of existing methods in describing the network heterogeneity, this paper defines the structure entropy based on global network topology while overcoming maximum flow bottleneck in the network, adds a medial measure considering the series-parallel structure and proposes a new network structure entropy—SP structure entropy (series-parallel structure entropy). On the one hand, through the analysis of special networks, we show that the entropy indicator proposed in this paper has advantages in considering the importance of series-parallel nodes and can overcome the deficiency of other network structure entropies to a certain extent; on the other hand, through theoretical analysis and simulation experiments on typical networks, such as a regular network, random network, scale-free network and small-world network, we further prove that SP structure entropy is effective and usable in describing general networks.

## 2. Materials and Methods

### 2.1. SP Structure Entropy Model

Given an unweighted undirected network G(V,E), where the set of nodes $V=\{v_{1},v_{2},\cdots ,v_{r}\}$ consists of *r* nodes and the set of edges $E=\{e_{1},e_{2},\cdots ,e_{t}\}$ consists of *t* edges, in order to avoid the difference in analysis caused by whether the network has self-loop or not, this paper assumes that node can reach itself in 0 step. The centrality metric of node *k* considering the series-parallel structure between the node pair $\left(v_{i},v_{j}\right)$ in the network is defined as

(1) $$\left\{\begin{array}{c} b_{k}\left(i,j\right)=\frac{\left|p_{ij}^{k}\right|}{\left|p_{ij}\right|}\cdot \sum_{p_{ij}^{k}}^{}\frac{1}{\left|c_{ij}^{k}\right|},\;p_{ij}\neq \emptyset \\ b_{k}\left(i,j\right)=0,\;p_{ij}=\emptyset \end{array}\right.$$

where ∅ denotes an empty set; $p_{ij}$ denotes the set of maximal flow possible paths between nodes $v_{i}$ and $v_{j}$; $\left|p_{ij}\right|$ denotes the number of paths in $p_{ij}$; $p_{ij}^{k}$ denotes the set of paths through node $v_{k}$ in the maximum flow possible path between nodes $v_{i}$ and $v_{j}$; $\left|p_{ij}^{k}\right|$ denotes the number of paths in $p_{ij}^{k}$; and $c_{ij}^{k}$ denotes the set consisting of the number of path nodes in $p_{ij}^{k}$, $v_{i}\neq v_{j}\neq v_{k}$.

From Equation (1), it can be seen that $\frac{\left|p_{ij}^{k}\right|}{\left|p_{ij}\right|}$ is a measure of whether node $v_{k}$ is in a parallel structure and its contribution to the parallel structure, and $\sum_{p_{ij}^{k}}^{}\frac{1}{\left|c_{ij}^{k}\right|}$ is a measure of whether node $v_{k}$ to the series structure in which it is located. To effectively measure parallel structures in the network, we only include the node pairs $\left(v_{i},v_{j}\right)$ with $\left|p_{ij}\right|\geq 1$ in the consideration range of $b_{k}\left(i,j\right)$.

**Figure 2:**
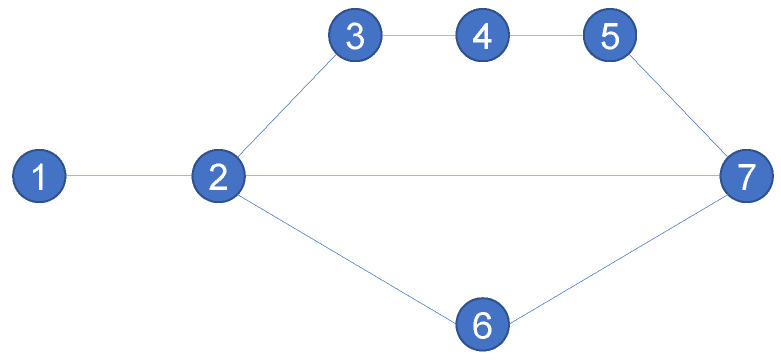
A 0-1 network with a series-parallel structure given source node 1 and sink node 7, where nodes 3, 4 and 5 are in the same series structure.

In order to deepen the understanding of Equation (1), Figure 2 is given, where nodes 3, 4 and 5 are in the same series structure as shown, we illustrate Figure 2: take node 4 between node pair (1,7) as an example, $p_{17}=\left\{\left(1,2,3,4,5,7\right),\left(1,2,7\right),\left(1,2,6,7\right)\right\}$, $p_{17}^{4}=\left(1,2,3,4,5,7\right)$; therefore, $\frac{\left|p_{17}^{4}\right|}{\left|p_{17}\right|}=\frac{1}{3}$. The number of nodes on path (1,2,3,4,5,7) in the set $p_{17}$ is 6, $\left|p_{17}^{4}\right|$. In the end, $\frac{\left|p_{17}^{4}\right|}{\left|p_{17}\right|}\cdot \sum_{p_{17}^{4}}^{}\frac{1}{\left|c_{17}^{4}\right|}=\frac{1}{3}\cdot \frac{1}{6}=\frac{1}{18}$.

Since the node pairs satisfying $\left|p_{ij}\right|\geq 1$ are not unique, in practice, we average $b_{k}$ over all node pairs $\left(v_{i},v_{j}\right)$ that satisfy the condition:

(2) $$b_{k}^{\prime }=\frac{\sum_{\left|p_{ij}\right|\geq 1}^{}b_{k}\left(i,j\right)}{B}$$

where *B* is the number of node pairs $\left(v_{i},v_{j}\right)$ in the network that satisfy $\left|p_{ij}\right|\geq 1$.

Define $d_{k}^{\prime }$ as the degree value of node $v_{k}$ in the undirected network, and then $a_{ij}$ represents the element value of the *i*th row and the *j*th column in the adjacency matrix of network *G*.

(3) $$d_{k}^{\prime }=\sum_{i}a_{ki}$$

As Borgatti and Everett clearly indicated when summarizing node centrality from the perspective of graph theory [20], the essence of a node centrality measure is to evaluate the participation of nodes in a network walk structure: the measure similar to the degree centrality is the radial measure, and the measure similar to the betweenness is the medial measure.

Therefore, $b_{k}^{\prime }$ is a typical medial measure method, which reflects the dependence of series-parallel structure on node $v_{k}$, measures the number of paths through node $v_{k}$ in parallel structure and the importance of being allocated by node $v_{k}$ in series structure. $d_{k}^{\prime }$ is a typical radial measure, a centrality measure method that takes a given node as the starting node or end node in a network path, essentially reflecting the connection between node $v_{k}$ and other nodes in network [14]. $b_{k}^{\prime }$ and $d_{k}^{\prime }$ constitute two parts of network walk position classification considering a series-parallel structure [20].

Radial and medial measures reflect the different roles played by nodes in a network and jointly determine the participation of nodes. In particular, this paper considers the influence of a series-parallel structure on the node importance in a medial measure. The two complement each other to form the total contribution of a given node to the network, which more comprehensively describes the node importance and network heterogeneity.

Radial and medial measures also have corresponding meanings in the field of social science: The former reflects bonding social capital—that is, from the perspective of internal connection, solidarity and trust, it is argued that social capital is the connection between individuals within an organization and small groups, which is used to reflect the connection of homogeneous groups. The latter reflects bridging social capital, from the perspectives of external relations, information asymmetry and power interests, and it is believed that the value of social capital originates from participating in and controlling the diffusion of information, which is used to reflect the ties of heterogeneous groups [20,21].

Similarly, from the perspective of social capital, aggregating social capital and outreach social capital are two aspects of individual social capital, which are indispensable and should be considered in a unified way [14]. Considering radial and medial measures comprehensively, the node importance of node $v_{k}$ can be defined as

(4) $$I_{k}^{\prime }=\alpha b_{k}^{\prime }+\beta d_{k}^{\prime }$$

where α and β are the weights of medial and radial measures, respectively, α+β=1,0≤α,β≤1. α=1,β=0 indicates the extent to which a given node contributes to the network considered from the medial measure only; conversely, α=0,β=1 means that only the radial measure is considered, and accordingly, $I_{k}^{\prime }$ degenerates into a local measurement.

Therefore, the relative importance of node $v_{k}$ in the network can be defined as

(5) $$I_{k}=\frac{I_{k}^{\prime }}{\sum_{n=1}^{N}I_{n}^{\prime }}=\frac{\alpha b_{k}^{\prime }+\beta d_{k}^{\prime }}{\sum_{n=1}^{N}\left(\alpha b_{n}^{\prime }+\beta d_{n}^{\prime }\right)}=\frac{\frac{e_{m}^{T}M_{v_{i},v_{j}}^{v_{k}}e_{m}}{m}+\frac{\beta }{\alpha }e_{N}^{T}A^{T}\delta_{k}}{\sum_{n=1}^{N}\left(\frac{e_{m}^{T}M_{v_{i},v_{j}}^{v_{n}}e_{m}}{m}+\frac{\beta }{\alpha }e_{N}^{T}A^{T}\delta_{n}\right)}$$

where $M_{v_{i},v_{j}}^{v_{k}}$ is a m×m dimension matrix composed of $b_{k}$ calculated by Equation (1) for all node pairs $\left(v_{i},v_{j}\right)$ satisfying the parallel structure conditions of node $v_{k}$, and *m* is the number of node pairs that satisfy the condition, that is, m=S(k). Similarly, *m* in the denominator is the number of node pairs that satisfy the parallel structure condition of node $v_{n}$. $e_{m}$ is a *m*-dimensional column vector with element 1. $e_{m}^{T}$ is the transpose of $e_{m}$. *A* is the adjacency matrix of network *G*. $A^{T}$ is the transpose of *A*. *N* is the network size, and $\delta_{k}$ is a *N*-dimensional column vector, where the *k*th element is 1 and the others are 0.

In Equation (5), $\frac{\beta }{\alpha }$ is a constant. This paper holds that a medial measure is as important as a radial measure, $\frac{\beta }{\alpha }=1$, that is,

(6) $$I_{k}≃\frac{e_{m}^{T}M_{v_{i},v_{j}}^{v_{k}}e_{m}+me_{N}^{T}A^{T}\delta_{k}+△}{\sum_{n=1}^{N}\left(e_{m}^{T}M_{v_{i},v_{j}}^{v_{n}}e_{m}+△\right)+me_{N}^{T}A^{T}e_{N}}$$

To avoid the calculation of the above equation being meaningless when V≠∅ and E=∅, the △ term is introduced to satisfy $△∼O(\frac{1}{N^{2}}),N>1$. The introduction of this term does not affect the results of the network structure heterogeneity analysis.

Based on Equation (6), the entropy of network structure in this paper is defined as

(7) $$\begin{array}{cc} H & =-\sum_{k=1}^{N}I_{k}\log I_{k}\\ \; & =-\sum_{k=1}^{N}\frac{e_{m}^{T}M_{v_{i},v_{j}}^{v_{k}}e_{m}+me_{N}^{T}A^{T}\delta_{k}+△}{\sum_{n=1}^{N}\left(e_{m}^{T}M_{v_{i},v_{j}}^{v_{n}}e_{m}+△\right)+me_{N}^{T}A^{T}e_{N}}\times \log\frac{e_{m}^{T}M_{v_{i},v_{j}}^{v_{k}}e_{m}+me_{N}^{T}A^{T}\delta_{k}+△}{\sum_{n=1}^{N}\left(e_{m}^{T}M_{v_{i},v_{j}}^{v_{n}}e_{m}+△\right)+me_{N}^{T}A^{T}e_{N}} \end{array}$$

where $v_{i},v_{j}\in S\left(k\right),S\left(k\right)=\left\{\left(v_{i},v_{j}\right):1\leq v_{i}\leq N;1\leq v_{j}\leq N;v_{i}\neq v_{j}\neq v_{k}\right\}$.

SP structure entropy can not only consider the network heterogeneity from a global perspective but also consider the influence of the series-parallel structure on node importance. The network shown in Figure 1a, Table 1 gives the calculation results of the node importance $I_{k}$ of Wu structure entropy, SD structure entropy, FB structure entropy and SP structure entropy. It can be seen that Wu structure entropy and SD structure entropy have no ability to distinguish node importance with the same degree value, even though node 2 and nodes 4, 5, which are in entirely different series-parallel situations in the network, as long as their degree values are the same, have the same node importance in the network, which clearly does not conform to the actual situation.

Under the measurement of FB structure entropy and SP structure entropy, the importance of node 2 and nodes 3, 4 in the network can be distinguished, which is in line with the actual situation. To illustrate the case of series structure, we calculate the network structure entropies in Figure 2, and the results are shown in Table 2: it can be seen that nodes 3, 4 and 5 and 6 have the same degree value, although they are all in parallel structure, and nodes 3, 4 and 5 are in different series structure from node 6. For this, Wu structure entropy and SD structure entropy cannot distinguish the difference; however, FB structure entropy and SP structure entropy can distinguish this structural difference.

Since the ineffectiveness of Wu and SD structure entropies in series-parallel structure has been proven, we only make further statements for FB and SP structure entropies. For Figure 1a, the node importance results under FB and SP structure entropies from the whole network are shown in Table 1, and the corresponding results of Figure 1b are shown in Table 3.

It can be seen that both FB and SP structure entropies can reflect the fact that, after adding node 7 between node 3 and node 6 (that is, adding a parallel path between node pair (3, 6)), the importance of other parallel paths between node pair (3, 6) is shared. Specifically, from Figure 1a to Figure 1b, the importance of node 4 changes from 0.08 to 0.0541 under the FB structure entropy measure and from 0.1433 to 0.1041 under the SP structure entropy measure. The importance of node 4 is allocated and becomes smaller after the addition of node 7. Therefore, the validity of FB structure entropy and SP structure entropy is verified for the whole network heterogeneity.

Since both FB structure entropy and SP structure entropy add the node degree value as the measurement standard, it is impossible to determine whether the changes in node importance are caused by the change of node degree value or the change of network structure. In view of this, we examine the absolute importance of nodes between a single node pair, that is, we calculate according to $I_{k}^{\prime }=\alpha b_{k}^{\prime }+\beta d_{k}^{\prime }$, where $b_{k}^{\prime }$ is radial measure of structure entropy and $d_{k}^{\prime }$ is medial measure, α+β=1. Specifically, the node pair (1, 6) in Figure 2 is selected as the consideration object. The importance of node 4 in the structure of node pair (1, 6) is calculated, and the results are shown in Table 4.

As can be seen from Table 4, the absolute importance under the FB structure entropy measurement has not changed. In contrast, the SP structure entropy has changed, indicating that FB structure entropy does not reflect the structural change of node pair (1, 6) after adding node 7; however, the SP structure entropy can reflect the change. To better understand, the specific calculation processes of FB structure entropy and SP structure entropy are shown and analyzed next.

### 2.2. A Detailed Explanation of SP Structure Entropy Model

#### 2.2.1. FB Structure Entropy

First, the maximum flow matrix of the corresponding network in Figure 1a is calculated as

$$W=\left[\begin{array}{cccccc} 0 & 1 & 1 & 1 & 1 & 1\\ 1 & 0 & 1 & 1 & 1 & 1\\ 1 & 1 & 0 & 2 & 2 & 3\\ 1 & 1 & 2 & 0 & 2 & 2\\ 1 & 1 & 2 & 2 & 0 & 2\\ 1 & 1 & 3 & 2 & 2 & 0 \end{array}\right]$$

It can be seen that the maximum network flow value between node pairs (1, 6) is 1. Since the importance of node 4 is considered, the maximum flow matrix of the network is recalculated after removing node 4 from the network as

$${}^{4}W^{*}=\left[\begin{array}{ccccc} 0 & 1 & 1 & 1 & 1\\ 1 & 0 & 1 & 1 & 1\\ 1 & 1 & 0 & 2 & 2\\ 1 & 1 & 2 & 0 & 2\\ 1 & 1 & 2 & 2 & 0 \end{array}\right]$$

The maximum flow value between node pair (1, 6) is still 1.

Therefore, in Figure 1a, the absolute node importance to node 4 in the node pair (1, 6) structure is

$$I_{4(a)}^{\prime }=\alpha b_{4}^{\prime }+\beta d_{4}^{\prime }=b_{4}^{\prime }+d_{4}^{\prime }=1-1+2=2$$

For Figure 1b, the network maximum flow matrix is calculated as

$$W=\left[\begin{array}{ccccccc} 0 & 1 & 1 & 1 & 1 & 1 & 1\\ 1 & 0 & 1 & 1 & 1 & 1 & 1\\ 1 & 1 & 0 & 2 & 2 & 4 & 2\\ 1 & 1 & 2 & 0 & 2 & 2 & 2\\ 1 & 1 & 2 & 2 & 0 & 2 & 2\\ 1 & 1 & 4 & 2 & 2 & 0 & 2\\ 1 & 1 & 2 & 2 & 2 & 2 & 0 \end{array}\right]$$

The maximum flow value between node pair (1, 6) is 1. Similarly, we recompute the maximum flow matrix after removing node 4 as

$${}^{4}W^{*}=\left[\begin{array}{cccccc} 0 & 1 & 1 & 1 & 1 & 1\\ 1 & 0 & 1 & 1 & 1 & 1\\ 1 & 1 & 0 & 2 & 3 & 2\\ 1 & 1 & 2 & 0 & 2 & 2\\ 1 & 1 & 3 & 2 & 0 & 2\\ 1 & 1 & 2 & 2 & 2 & 0 \end{array}\right]$$

After recalculation, the maximum flow value between node pair (1, 6) remains at 1.

Therefore, in Figure 1b, the absolute node importance to node 4 in the node pair (1, 6) structure is

$$I_{4(b)}^{\prime }=\alpha b_{4}^{\prime }+\beta d_{4}^{\prime }=b_{4}^{\prime }+d_{4}^{\prime }=1-1+2=2$$

It can be seen that $I_{4(a)}^{\prime }=I_{4(b)}^{\prime }$, and the FB structure entropy cannot reflect the change of parallel structure between node 1 and node 6. At its root, FB structure entropy is a measure of the structure entropy based on the network maximum flow as can be seen from Figure 1a,b. Whether node 7 is added or not, the maximum flow value between node 1 and node 6 is limited by the capacity of path (1, 3)—that is, in a 0-1 network, the maximum flow between node pair (1, 6) is 1. Therefore, no matter how many nodes are added in parallel with node 4, FB structure entropy cannot reflect that the importance of node 4 is shared by its parallel paths. Therefore, FB structure entropy cannot reflect the change of network structure between some specific node pairs.

#### 2.2.2. SP Structure Entropy

We calculate the importance of node 4 between node pair (1, 6) in Figure 1a. The set of maximum flow possible paths between node pair (1, 6) is $p_{16}=\{\left(1,2,3,4,6\right),\;\left(1,2,3,6\right),$(1,2,3,5,6)} and the set of paths passing through node 4 in set $p_{16}$ is $p_{16}^{4}=\left\{\left(1,2,3,4,6\right)\right\}$. Then, the number of paths in $p_{16}$ is $\left|p_{16}^{4}\right|=3$, and the number of paths in $p_{16}^{4}$ is $\left|p_{16}^{4}\right|=1$. The number of nodes on the path (1,2,3,4,6) is 5; thus, $\left|c_{16}^{4}\right|=5$. Therefore, the medial measure of the importance of node 4 on the node pair (1, 6) structure in Figure 1a is

$$b_{4}^{\prime }=\frac{\left|p_{16}^{4}\right|}{\left|p_{16}\right|}\cdot \sum_{p_{16}^{4}}^{}\frac{1}{\left|c_{16}^{4}\right|}=\frac{1}{3}\cdot \frac{1}{5}=\frac{1}{15}=0.0667$$

Therefore, the absolute importance of node 4 in the node pair (1, 6) structure in Figure 1a is

$$I_{4(a)}^{\prime }=\alpha b_{4}^{\prime }+\beta d_{4}^{\prime }=b_{4}^{\prime }+d_{4}^{\prime }=0.0667+2=2.0667$$

Next, calculating the importance of node 4 between node pair (1,6) in Figure 1b. The set of paths between node pair (1, 6) is $p_{16}=\left\{\left(1,2,3,4,6\right),\left(1,2,3,6\right),\left(1,2,3,5,6\right),\left(1,2,3,7,6\right)\right\}$, and the paths set through node 4 in $p_{16}$ are $p_{16}^{4}=\left\{\left(1,2,3,4,6\right)\right\}$. Then, $\left|p_{16}\right|=4,\left|p_{16}^{4}\right|=1,\left|c_{16}^{4}\right|=5$. Therefore, the medial measure of the importance of node 4 on the node pair (1, 6) structure in Figure 1b is

$$b_{4}^{\prime }=\frac{\left|p_{16}^{4}\right|}{\left|p_{16}\right|}\cdot \sum_{p_{16}^{4}}^{}\frac{1}{\left|c_{16}^{4}\right|}=\frac{1}{4}\cdot \frac{1}{5}=\frac{1}{20}=0.05$$

Therefore, the absolute importance of node 4 in the node pair (1, 6) structure in Figure 1b is

$$I_{4(b)}^{\prime }=\alpha b_{4}^{\prime }+\beta d_{4}^{\prime }=b_{4}^{\prime }+d_{4}^{\prime }=0.05+2=2.05$$

It can be seen that $I_{4(a)}^{\prime }\neq I_{4(b)}^{\prime }$, and thus SP structure entropy can reflect the change of parallel structure between node pair (1, 6).

The above analysis shows that both FB structure entropy and SP structure entropy can reflect the change of network heterogeneity through node importance when considering the whole network structure. However, when focusing on a specific node pair structure, FB structure entropy cannot accurately reflect the corresponding heterogeneity changes due to the maximum flow. In contrast, SP structure entropy considers the series-parallel structure in the network; therefore, it can accurately describe the heterogeneity changes, both in the whole network structure and in the local node pair structure.

## 3. Results and Discussion

### 3.1. Structure Entropy Simulation Experiments for Typical Networks

In general, network models can be divided into three categories [22]: the first category is random networks, such as the ER random network proposed by Erdos and Renyi [23]; the second is regular networks, such as a star network and nearest-neighbor coupled network [22]; and the third type is the network structure between random and regular networks, which combines some characteristics of both regular and random networks, such as the BA scale-free network proposed by Barabasi and Albert [24] and WS small-world network proposed by Watts and Strogatz [25]. We constructed simulations of typical social networks, a nearest-neighbor coupled network, a star network, an ER random network, a BA scale-free network and a WS small-world network, and we calculated their structure entropies.

It is worth noting that network heterogeneity is a context-dependent concept, for instance, in the BA scale-free network model, degree centrality is a commonly used metric for heterogeneity and is widely used in immunization strategy formulation [26]; however, it performs poorly in other situations where the global network structure needs to be considered. Thus, the significance of the experiment is to show that each structure entropy metric can be effective in heterogeneity measurements of typical networks while maintaining its own superiority in specific contexts. The simulation experiments were implemented with Python3.8 programming.

To facilitate comparison with other typical network entropies, the typical networks chosen for the experiments were all undirected and unweighted; to facilitate the comparison between different network entropies, and the natural logarithm was used for all numerical calculations of entropy in the experiments. As △ can take any small non-zero number, we set △ to $10^{-9}$.

#### 3.1.1. Simulation Experiment of Typical Social Network Structure Entropy

**Figure 3:**
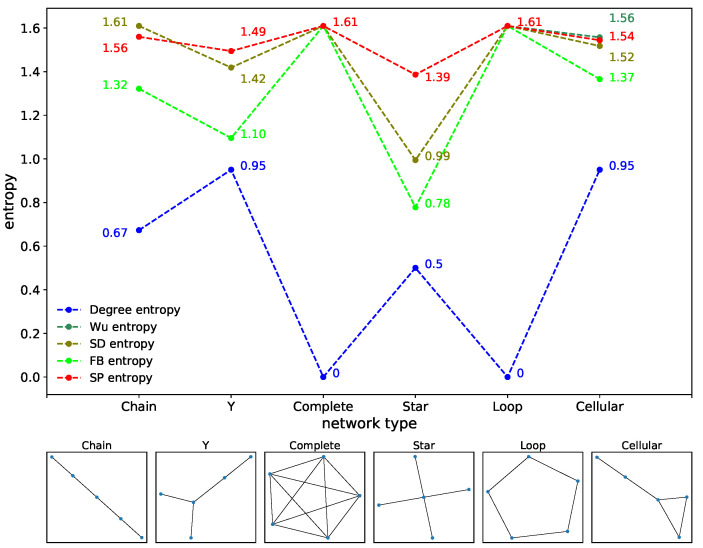
Structure entropy and topology structure of typical social networks. The size of typical social networks is 5.

In this paper, six typical social network structures were selected for structure entropy simulation, namely the chain network, Y network, complete network, star network, loop network and cellular network. We calculated the degree distribution entropy, Wu structure entropy, SD structure entropy, FB structure entropy and SP structure entropy for each network, and the results are demonstrated in Table 5. To present the results more visually, the corresponding line diagram and network topology are shown in Figure 3.

It can be seen that, since degree distribution entropy only considers the distribution function of node degrees, which is 0 in the network with the same node degrees, it shows an opposite trend to the other structure entropies, while the rise and fall trend of other structure entropies between different networks is the same. In particular, accurate characterization of the star network, has always been a reflection of the superiority of SD and FB structure entropies, and SP structure entropy can also solve this problem well. The minimum value of structure entropy of six social networks under the same size is located at the star network, and the strong heterogeneity of the star network is confirmed [11]. Therefore, SP structure entropy can effectively reflect the heterogeneity of typical social networks and is particularly similar to the calculation results of Wu structure entropy.

#### 3.1.2. Simulation Experiment of Typical Network Structure Entropy

In this paper, five typical networks (nearest-neighbor coupled network, star network, ER random network, BA scale-free network and WS small-world network) are numerically simulated, respectively, and the degree distribution entropy, Wu structure entropy, SD structure entropy, FB structure entropy and SP structure entropy are compared to prove that SP structure entropy is effective and accurate in judging the overall heterogeneity of typical networks on the basis of maintaining sensitivity to the series-parallel structure.

**Figure 4:**
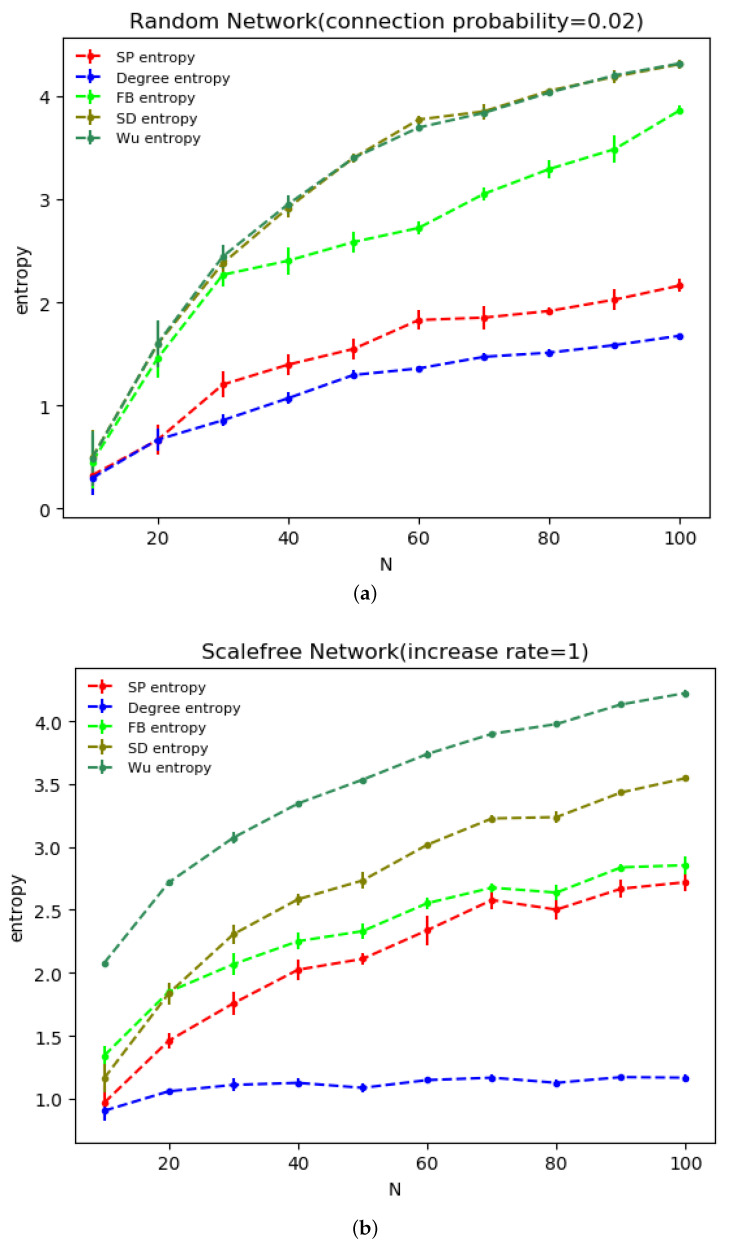
Structure entropy of the ER random network and BA scale-free network under different structure entropy indicators. (**a**) shows the results for ER random network with connection probability q=0.02, (**b**) shows the results for BA scale-free network with growth rate u=1. Each result is independently performed 10 times and then averaged, and the result curves are shown with error bars.

Figure 4 shows the structure entropies of the ER random network with connection probability q=0.02 (network density is 0.02) and BA scale-free network with growth rate u=1 under different structure entropy measurements. Each indicator was independently performed 10 times and then averaged. From Figure 4a, it can be seen that the evolutionary process of the ER random network reflects a significant entropy increase effect under all five structure entropy indicators, in which the curves of the SD and Wu structure entropies almost overlap. While the two types of network entropy evolve in very similar ways, the meanings expressed are very different.

For example, when adding the first edge to the network, Wu structure entropy only considers the two connected nodes, that is, the entire network consists of only two edges, and the entropy value takes the maximum value ln2. This entropy value appears to be small throughout the network evolution process due to the network size effect. In contrast, SD structure entropy considers the entire network structure. Network heterogeneity is strong when there is only one edge in the network, and the difference between nodes is large. When the network size is 30, the trend of FB structure entropy and SD structure entropy diverges, which, due to FB structure entropy, includes not only the radial measure based on node degree value but also the medial measure based on the maximum flow. As the network size increases and there are more connections between nodes, the medial measure comes into play.

It can be seen that the value of degree distribution entropy is the smallest, which also verifies that, under the degree distribution entropy indicator, the network takes longer to reach a steady state and the entropy value grows significantly slower with network evolution than with the other network structure entropies [14]. The SP structure entropy curve lies above degree distribution entropy and below SD, FB and Wu structure entropies in Figure 4, that is, SP structure entropy is greater than the degree distribution entropy and smaller than the SD, FB and Wu structure entropies, which also indicates that SP structure entropy, consisting of radial and medial measures, considers more medial measures based on network series-parallel structure than degree distribution entropy, which only considers the degree distribution.

Thus, after the network size exceeds 20, SP structure entropy is greater than the degree distribution entropy due to the presence of series-parallel paths in the network. Since medial measure of SP structure entropy considers series-parallel paths in the network, the importance of a node on the series-parallel path will be shared by other nodes, and thus the SP structure entropy is smaller than the SD, FB and Wu structure entropies that do not consider series-parallel paths, thus, showing that the SP structure entropy has a higher sensitivity to series-parallel structure in the network compared to other structure entropy indicators.

The BA scale-free network illustrated in Figure 4b shows a significant entropy increase effect with the evolution of network under five network entropy indicators. Precisely, similar to the results for ER random network, the maximum and minimum structure entropy values remain Wu structure entropy and degree distribution entropy. Different from the ER random network, the changing trend of the SD structure entropy curve is still synchronized with Wu structure entropy, however, is no longer numerically equivalent to it; the entropy increase effect of degree distribution entropy is not obvious. Particularly after the network size reaches 20, the degree distribution entropy does not change greatly.

The former indicates that both SD structure entropy and Wu structure entropy can more accurately reflect the heterogeneity of the ER random network and BA scale-free network; however, there will be a gap in the numerical results due to the difference in the scope of investigation; the latter shows that, under the measurement of degree distribution entropy, the BA scale-free network reaches a steady state earlier than other structure entropy indicators, reflecting a slight lack of degree distribution entropy in portraying network heterogeneity [14].

The FB structure entropy and SP structure entropy curves are located in the middle, and the SP structure entropy value is slightly smaller than FB structure entropy due to the sharing effect of series-parallel structure on node importance being considered. However, the trend of the two is roughly the same with the increasing network size, which also reflects the fact that the parallel structure measure of SP structure entropy is weakened due to the structure characteristics of the BA scale-free network, thus, showing a similar growth rate change trend to that of FB structure entropy.

**Figure 5:**
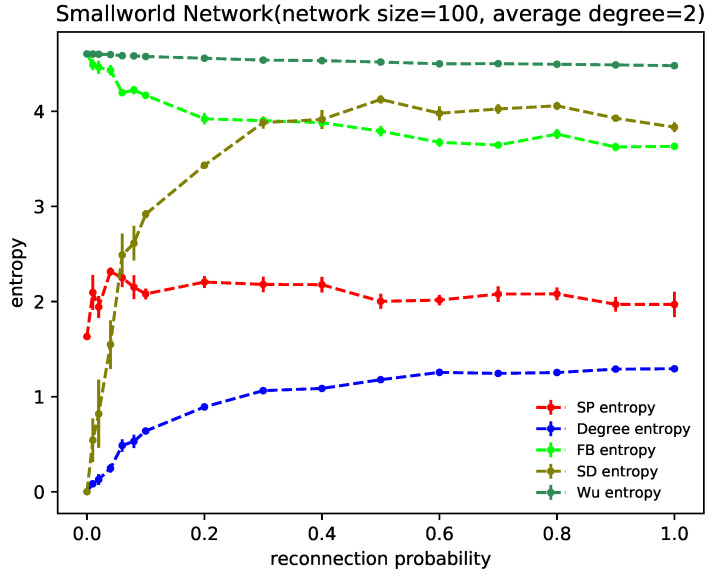
Structure entropy of the WS small-world network under different structure entropy indicators, where the network size is 100 and the average degree is 2. Each result is independently performed 10 times and then averaged. The result curves are shown with error bars.

Figure 5 shows the structure entropy results of the WS small-world network with reconnection probability *p*. Each metric was independently performed 10 times and then averaged, where the network size is 100 and the average degree is 2. The evolution process of the network with reconnection probability *p* from 0 to 1 reflects the change of nearest-neighbor coupled network to random network. Wu structure entropy argues that network heterogeneity changes little during the evolution process, while SD structure entropy has a significant change in network heterogeneity. SP structure entropy, FB structure entropy and degree distribution entropy are intermediate between Wu structure entropy and SD structure entropy, all with a small range of variation.

It can be seen that SP structure entropy, SD structure entropy, FB structure entropy and degree distribution entropy all show noticeable changes at p∈[0,0.1]. Among them, SD structure entropy reaches a steady state after p=0.4. However, the sharp shift in entropy value still occurs before p=0.1, which also verifies that the small-world characteristics of the WS small-world network are mainly reflected in an interval of reconnection probability of [0,0.1] [25].

The small-world characteristics are mainly reflected in high clustering coefficients and short path lengths, except for Wu structure entropy, which is always in a steady state. The fastest curve into a steady state is the one corresponding to SP structure entropy. According to the construction idea of SP structure entropy, the series-parallel structures in the network do not contain only the shortest paths. From this perspective, SP structure entropy considers that small-world characteristics do not greatly change the network heterogeneity.

**Figure 6:**
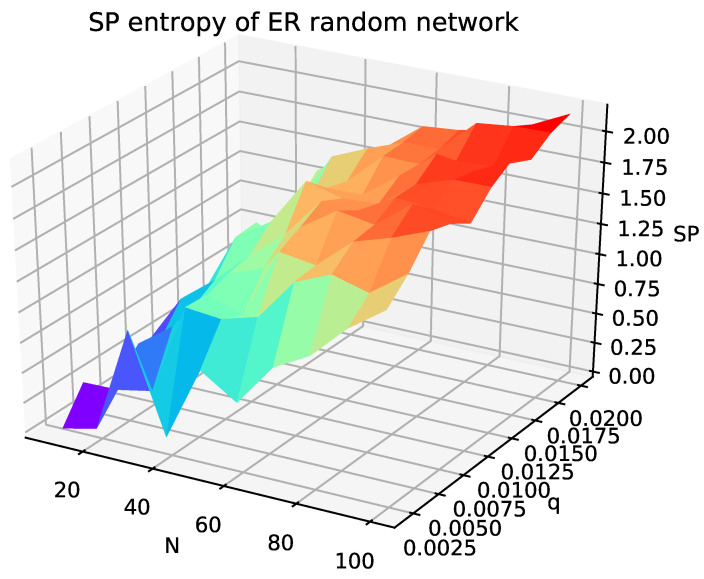
SP structure entropy of the ER random network, where the network size is increased from 10 to 100 in steps of 10, and the connection probability *q* is increased from 0.002 to 0.02 in steps of 0.002.

Figure 6 illustrates the SP structure entropy of the ER random network as the network size *N* and connection probability *q* change, where the ER random network is constructed according to N=[10,20,30,40,50,60,70,80,90,100] and q=[0.002,0.004,0.006,0.008,0.01,0.012,0.014,0.016,0.018,0.02]. As network size *N* increases, the SP structure entropy value of the ER random network increases substantially, and network size has a clear influence on the SP structure entropy. However, when the network size is fixed, the change of connection probability *q* does not cause a regular change in SP structure entropy, that is, the SP structure entropy values for connection probabilities in the range [0.002,0.02] remain in a stable range. Therefore, it can be concluded that, when the network connection probability is small, SP structure entropy is not affected by the change of network connection probability.

To further verify the validity and superiority of SP structure entropy, numerical simulations were conducted on four typical networks, namely the nearest-neighbor coupled network, star network, ER random network and BA scale-free network, and each index was independently performed 10 times and then averaged. Among them, the network evolution scale of the BA scale-free network was increased from 10 to 15 with a step size of 1, and the growth rate was constructed according to the maximum network density under the corresponding size. The ER random network was constructed according to the scale and density of the BA scale-free network in the evolution process, that is, the BA scale-free network and the ER random network have the same size and density. Table 6 shows the simulation sizes, growth rates and densities for the BA scale-free and ER random networks. The nearest-neighbor coupled network and star network were constructed according to the corresponding BA scale-free network size.

**Figure 7:**
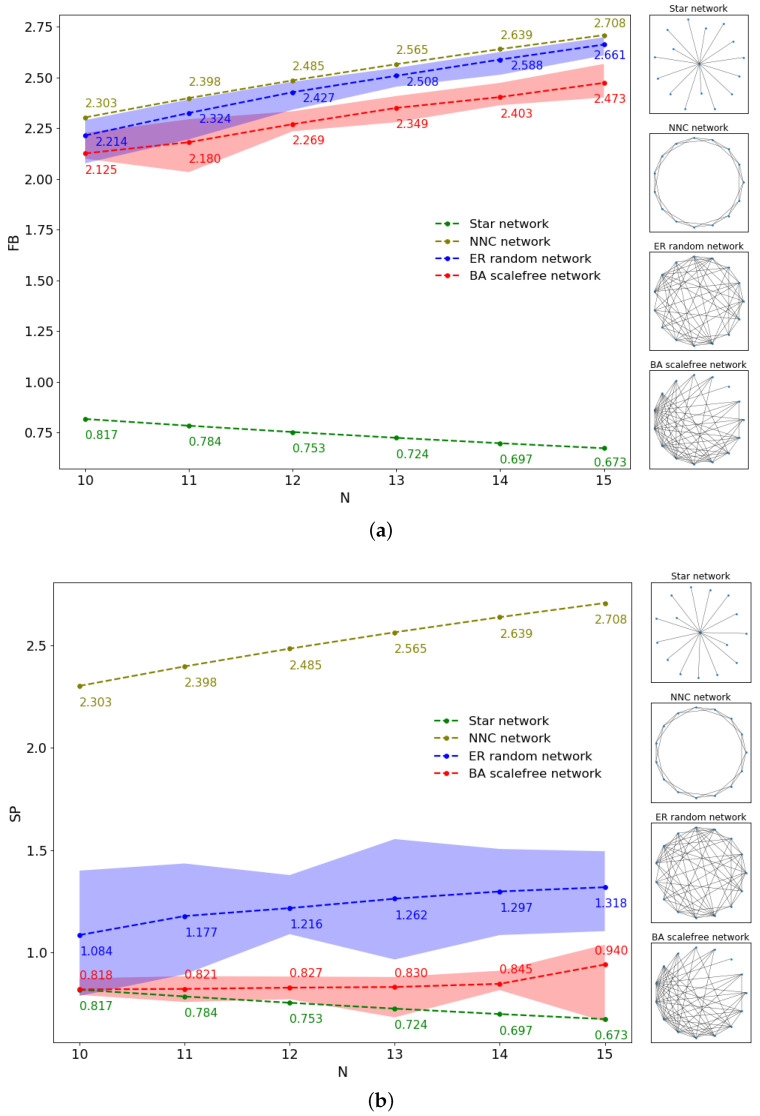
The structure entropy and topology structure of four typical networks. (**a**) the FB structure entropy and (**b**) the SP structure entropy. The results of the ER random network and BA scale-free network are independently performed 30 times and then averaged for the result curves with upper and lower bound regions. The network size *N* is increased from 10 to 15 in steps of 1.

Since the literature [14] verified that FB structure entropy is more effective than degree distribution entropy, Wu structure entropy and SD structure entropy on these four typical networks, we only compared SP structure entropy with FB structure entropy. Figure 7 shows FB and SP structure entropies for the nearest-neighbor coupled network, star network, ER random network and BA scale-free network. Since the ER random network and BA scale-free network were averaged over 30 experiments, we show the result curves with upper and lower bound regions.

The structure entropy of nearest-neighbor coupled network and star network are fixed for fixed network sizes, and we show the result curves without upper and lower bounds. It can be seen that both FB and SP structure entropies consider the bounds (maximum and minimum values) of network entropy correspond to the nearest-neighbor coupled network and star network, respectively, which is also consistent with the analysis in Section 3.1 and reflects the ability of SP structure entropy to accurately portray these two types of regular networks. According to the network structural characteristics, the BA scale-free network satisfies the characteristics that a large number of nodes have a small number of connections and a few nodes have a large number of connections; thus, its heterogeneity should be stronger than the ER random network under the same size and density.

Comparing the result curves of the ER random network and BA scale-free network in Figure 7, both the FB and SP structure entropies show that the BA scale-free network structure entropy is smaller than for the ER random network, which satisfies the network structure characteristics. However, as can be seen by the magnitude of difference, SP structure entropy can better distinguish between the BA scale-free network and ER random network in terms of the network heterogeneity.

### 3.2. Empirical Analysis

Previous studies have found that interpersonal relationship in enterprise is an essential factor affecting the performance of employees, and the location of employees in a social network can predict their personal performance [27,28]. A consulting network is a typical social network within an enterprise, which reflects an informal network relationship within an organization and is an important way for team members to exchange knowledge and information. These can effectively reflect interpersonal interactions. To further verify the practical value of SP structure entropy, we surveyed a small–medium enterprise in China engaged in airfreight, domestic logistics, rail-sea intermodal transport and international express.

**Figure 8:**
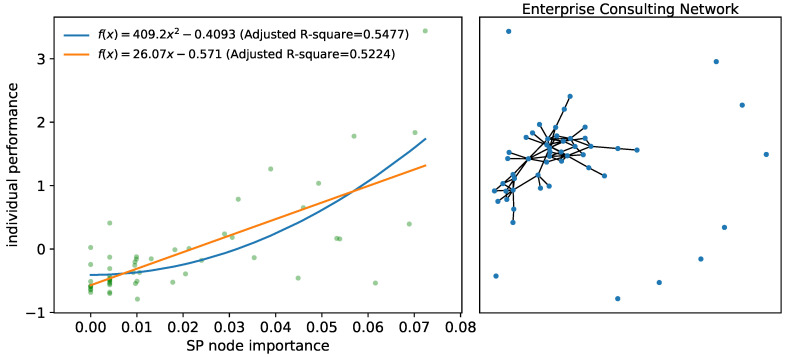
Regression results (**left**) and consulting network topology (**right**). The left shows the regression results with SP node importance as the independent variable and individual performance as the dependent variable. The right shows the consulting network of the enterprise, where the nodes represent employees.

From the perspective of the whole network, the research object is 52 employees of the enterprise. By analyzing their consulting network, we demonstrated the validity and superiority of node importance under SP structure entropy measurement (hereafter referred to as SP node importance, calculated by Equation (6)) in reflecting the location characteristics of the individual network, which in turn affects their personal performance. The network topology is shown in Figure 8 (right).

To examine the influence of SP node importance on individual performance, we used regression analysis to analyze the employee consulting network. The dependent variable of the regression model is the individual performance (denoted by *Z*), which is measured using wages and bonuses. The *Z*-score of average monthly income is calculated as in Equation (8), where *Y* is the actual income of an employee, μ is the average income of all employees and σ is the standard deviation of income. A *Z*-score of 0 means that the employee receives the average salary and bonus of the enterprise, while a *Z*-score of 1 means that the employee’s earnings are one standard deviation above the average income of the enterprise.

(8) $$Z=\frac{Y-\mu }{\sigma }$$

The independent variables are the SP node importance (denoted by SPNI), in-degree centrality (IDC), out-degree centrality (ODC), betweenness centrality (BC), in-degree closeness centrality (ICC) and out-degree closeness centrality (OCC). The control variables are gender, political affiliation and work time (years of service in the current position). First, a regression analysis was conducted with SP node importance as the independent variable, *Z*-score performance as the dependent variable and no other control variables considered. The results are shown in Figure 8 (left).

It can be seen that there is a significant positive correlation between individual performance and SP node importance, that is, the greater the SP node importance in the consulting network, the higher the corresponding individual performance. This is also consistent with the social significance that SP node importance as a good indicator of an individual’s absolute resource occupancy (node degree) and status specificity (whether or not they are in a series-parallel structure) in the network.

On the one hand, the node degree value considered by SP structure entropy can reflect an individual’s number of direct contacts. Based on social capital theory, the number of direct contacts can be regarded as a kind of social resource. Therefore, the greater the degree of value in the network, the more resources the individual obtains and the more critical the status is. On the other hand, as an important part of SP structure entropy, series-parallel structure reflects the special status of nodes in the network to a certain extent.

The more important the position of a node in the network information transmission, that is, the fewer serial nodes or parallel paths, the more special the node position in the network, and the greater the advantage of the corresponding individual acting as a competitor of the “information bridge”, the greater the opportunity to gain potential benefits and have advantages of information and control. Thus, by virtue of absolute resource occupation and status specificity, individuals with high SP node importance in the consulting network tend to achieve higher individual performance.

The above analysis demonstrated the validity of SP node importance in predicting individual performance. Next, we introduced other classical network indicators for comparison to prove the superiority of SP node importance. The regression analysis results are shown in Table 7. Models 1 to 6 used SPNI, IDC, ODC, BC, ICC and OCC as independent variables in the OLS regression model. As can be seen, each model is significant, justifying the reasonableness of using the social network to explain individual performance. Furthermore, the adjusted $R^{2}$ for model 1 is 0.594, which is the maximum value among all models, indicating that model 1 has the best fit.

Thus, compared with other network structure indicators, SP node importance has the optimum capacity in explaining individual performance. Among the control variables, political affiliation and work time also showed statistical significance: members of the Chinese Communist Party perform better than non-party members, likely because the party members are often strictly selected, which makes them better at their jobs as well; the longer employees work, the more comfortable they are in their job. Therefore, these two groups obtain better performance.

## 4. Conclusions

This paper proposed a new network structure entropy indicator-SP structure entropy by considering the network heterogeneity from a global perspective while considering the influence of the series-parallel structure on node importance. On the one hand, analyzing the network with series-parallel structure verified the superiority of SP structure entropy in considering the importance of series-parallel nodes, which can overcome the deficiency of other network structure entropies to a certain extent.

On the other hand, by analyzing and comparing the structure entropy of six typical social networks (such as the chain network, Y network and complete network) and five typical networks (such as the BA scale-free network, ER random network and WS small-world network), the validity of SP structure entropy in portraying general networks was verified.

We investigated an enterprise in China and demonstrated the superiority of SP node importance in reflecting network structure and explaining employee performance by analyzing the employee consulting network. Therefore, SP structure entropy as proposed in this paper revealed the characteristics of network series-parallel structure and at the same time portrayed the heterogeneity of different network structures, enriching the network structure indicator system.

The main work in this paper focused on the design of network structure entropy and the experimental analysis of its properties. More in-depth theoretical analysis and application will be the content of subsequent research. Specifically: The network structure entropy considering series-parallel structure proposed in this paper has a clear advantage for the network containing series-parallel structures, as the medial measure is based on the series-parallel structure and the radial measure is based on node degree distribution, while there should be multiple measurements of the node importance.

In the future, other measurements can be considered to be added to SP structure entropy so that it can also show advantages when measuring networks with insignificant series-parallel structures. In addition, a more brief mathematical analytic expression and quantitative description form of typical network structure entropy will be derived.

## Figures and Tables

**Table 1:** The difference of node importance of four structure entropies on the network of Figure 1a.

Node	Node Degree Value	Wu Structure Entropy	SD Structure Entropy	FB Structure Entropy	SP Structure Entropy
1	1	0.0714	0.2054	0.02	0.072
2	2	0.1428	0.0136	0.2	0.1447
3	4	0.2857	0.4109	0.44	0.2838
4	2	0.1428	0.0136	0.08	0.1433
5	2	0.1428	0.0136	0.08	0.1433
6	3	0.2142	0.3424	0.18	0.2125

**Table 2:** The difference of node importance of four structure entropies on the network of Figure 2.

Node	Node Degree Value	Wu Structure Entropy	SD Structure Entropy	FB Structure Entropy	SP Structure Entropy
1	1	0.0625	0.1176	0.0086	0.064
2	4	0.25	0.2941	0.2931	0.247
3	2	0.125	0.0882	0.1551	0.1255
4	2	0.125	0.0882	0.1551	0.1248
5	2	0.125	0.0882	0.1551	0.1263
6	2	0.125	0.0882	0.0344	0.1247
7	3	0.1875	0.2352	0.1982	0.1874

**Table 3:** The difference of node importance under two structure entropy measures on the network of Figure 1b.

Node	Node Degree Value	FB Structure Entropy	SP Structure Entropy
1	1	0.0135	0.0507
2	2	0.1622	0.1134
3	4	0.4459	0.2949
4	2	0.0541	0.1041
5	2	0.0541	0.1041
6	4	0.2162	0.2287
7	2	0.0541	0.1041

**Table 4:** The absolute importance of node 4 under two structure entropies.

	Figure 1a	Figure 1b
FB structure entropy	2	2
SP structure entropy	2.0667	2.05

**Table 5:** Structure entropy of six typical social network structures.

	Chain Network	Y Network	Complete Network	Star Network	Loop Network	Cellular Network
Degree distribution entropy	0.6730	0.9503	0	0.5004	0	0.9503
Wu structure entropy	1.5596	1.4942	1.6094	1.3863	1.3578	1.5571
SD structure entropy	1.6094	1.4185	1.6094	0.9944	1.6094	1.5175
FB structure entropy	1.3216	1.0956	1.6094	0.7777	1.6094	1.3655
SP structure entropy	1.5502	1.4764	1.6094	1.3578	1.6094	1.5455

**Table 6:** The network size, density and growth rate of the BA scale-free network and ER random network.

N	Network Increase Rate	Network Density
10	5	0.5556
11	5	0.5454
12	6	0.5454
13	6	0.5384
14	7	0.5384
15	7	0.5333

**Table 7:** The regression analysis results of the consulting network.

	Model 1	Model 2	Model 3	Model 4	Model 5	Model 6
Intercept term	−0.892 ***	−0.767 ***	−0.849 ***	−0.749 ***	−2.122 **	−1.596 ***
SPNI	24.072 ***					
IDC		0.149 ***				
ODC			0.1 ***			
BC				0.544 ***		
ICC					0.765 *	
OCC						0.435 **
gender	0.150	0.175	0.144	0.208	0.273	0.095
political affiliation	0.486 *	0.534 *	0.629 *	0.781 ***	0.659 *	0.644 *
work time	0.051 **	0.021 *	0.076 **	0.064 **	0.025 *	0.09 **
adjusted $R^{2}$	0.594	0.540	0.384	0.388	0.216	0.284
F-value	19.624 ***	15.940 ***	8.939 ***	9.067 ***	4.514 ***	6.062 ***

Note: The regression coefficients are shown in this table. In all models, the VIF indicator is far below the critical value of 10, and thus there is no significant multicollinearity. *** means *p* < 0.001, ** means *p* < 0.01, * means *p* < 0.05 (two-tailed test).

## Data Availability

Not applicable.

## Abbreviations

The following abbreviations are used in this manuscript:

SP	series-parallel
ER	Erdos and Renyi
BA	Barabasi and Albert
WS	Watts and Strogatz
SPNI	SP node importance
IDC	in-degree centrality
ODC	out-degree centrality
BC	betweenness centrality
ICC	in-degree closeness centrality
OCC	out-degree closeness centrality
OLS	ordinary least squares
